# LncRNA MALAT1 modulates ox-LDL induced EndMT through the Wnt/β-catenin signaling pathway

**DOI:** 10.1186/s12944-019-1006-7

**Published:** 2019-03-14

**Authors:** Hongrong Li, Qifei Zhao, Liping Chang, Cong Wei, Hongying Bei, Yujie Yin, Meng Chen, Hongtao Wang, Junqing Liang, Yiling Wu

**Affiliations:** 1 Hebei Medical University, No. 361, Zhongshan East Road, Changan District, Shijiazhuang, 050017 China; 2 National Key Laboratory of Luobing Research and Innovative Chinese Medicine, Shijiazhuang, 050035 China; 3 Hebei Key Laboratory of Luobing, Shijiazhuang, 050035 China; 4 Yiling Hospital of Hebei Medical University, The Key Laboratory of State Administration of Traditional Chinese Medicine, Shijiazhuang, 050091 China; 5 Hebei University of Chinese Medicine, Shijiazhuang, 050090 China

**Keywords:** Atherosclerosis, EndMT, Ox-LDL, MALAT1, Wnt/β-catenin

## Abstract

**Background:**

Endothelial-to-mesenchymal transition (EndMT) plays significant roles in atherosclerosis, but the regulatory mechanisms involving lncRNAs remain to be elucidated. Here we sort to identify the role of metastasis-associated lung adenocarcinoma transcript 1 (MALAT1) in ox-LDL-induced EndMT.

**Methods:**

The atherosclerosis model was established by feeding ApoE^−/−^ mice with high-fat diet, and the levels of lncRNA MALAT1 in mouse arterial tissue were detected by RT-qPCR. Cell model was established by treating human umbilical vein endothelial cells (HUVECs) with ox-LDL, and the levels of EndMT markers, such as CD31, vWF, α-SMA and Vimentin and lncRNA MALAT1 levels were detected and their correlations were analyzed. The role of MALAT1 in EndMT and its dependence on Wnt/β-catenin signaling pathway was further detected by knocking down or overexpressing MALAT1.

**Results:**

MALAT1 was upregulated in high-fat food fed *ApoE*^−/−^ mice. HUVECs treated with ox-LDL showed a significant decrease in expression of CD31 and vWF, a significant increase in expression of α-SMA and vimentin, and upregulated MALAT1. An increased MALAT1 level facilitated the nuclear translocation of β-catenin induced by ox-LDL. Inhibition of MALAT1 expression reversed nuclear translocation of β-catenin and EndMT. Moreover, overexpression of MALAT1 enhanced the effects of ox-LDL on HUVEC EndMT and Wnt/β-catenin signaling activation.

**Conclusions:**

Our study revealed that the pathological EndMT required the activation of the MALAT1-dependent Wnt/β-catenin signaling pathway, which may be important for the onset of atherosclerosis.

**Trial registration:**

Not applicable.

## Background

Atherosclerosis is a multistep cardiovascular disease promoted by several kinds of risk factors and is one of the most common causes of mortality in the world [1]. To date, the pathological mechanism responsible for the development and progression of atherosclerosis is largely unrevealed. Endothelial-to-mesenchymal transition (EndMT), a special type of epithelial-to-mesenchymal (EMT) [2], is a complex biological process through which endothelial cells convert to mesenchymal or myofibroblastic cells. EndMT is characterized by the loss of specific endothelial markers, and acquisition of mesenchymal markers. Recent studies have demonstrated that EndMT might be closely related to the atherosclerosis progression [3, 4]. For example, EndMT-derived cells are common in atherosclerotic lesions in mice [3]. The ‘transitioning’ cells co-expressing endothelial and fibroblast/ mesenchymal proteins are also detected in human plaques [5]. Moreover, EndMT enhances plaque calcification to increase plaque burden [6], and the extent of EndMT is associated with plaque instability in the clinical events [5]. More importantly, the canonical risk factors leading to atherosclerotic lesion formation, such as cytokines [7], reactive oxygen species (ROS) [8], and high glucose [9] are also found to trigger EndMT. As one of the causal risk factors for atherosclerosis, oxidized low-density lipoprotein (ox-LDL) activates the receptor of ox-LDL in endothelial cells [10] to up-regulate stimulators such as inflammatory cytokines [11], ROS [12] and TGF-β [13], which can further induce EndMT. However, the precise regulators of EndMT in response to ox-LDL are still poorly understood.

Metastasis associated lung adenocarcinoma transcript 1 (MALAT1), which is also known as NEAT2 (noncoding nuclear-enriched abundant transcript 2) and was first identified in 2003 in non-small cell lung cancer [14, 15], is one of the initially identified long non-coding RNAs associated with human diseases. MALAT1 is evolutionarily conserved among mammalians, and is abundantly expressed in many tissues [15, 16]. Mature MALAT1 is localized in nucleus with a cytoplasmic tRNA-like small RNA, known as mascRNA [17]. When RNA polymerase II-dependent transcription is activated, MALAT1 becomes enriched in nuclear speckles-dynamic and irregularly shaped nuclear domains involved in pre-mRNA splicing and RNA transport in mammalian cells [18]. MALAT1 has been implicated in functional regulation of a variety of cells involved in atherosclerosis, such as vascular endothelial cells [19–21], smooth muscle cells [22] and macrophages [23]. It is reported that ox-LDL could induce MALAT1 transcription through NF-κB pathway in THP-1-derived macrophages [24], and MALAT1 can modulate TGF-β1-induced EndMT in endothelial progenitor cells [25].

Wnt/β-catenin pathway, the canonical Wnt signaling, is reported to be involved in multiple aspects of the development and progression of atherosclerosis [26], including endothelial dysfunction [27], macrophage activation [28, 29] and the proliferation and migration of vascular smooth muscle cells [30]. On the other hand, the Wnt/β-catenin signaling pathway is reported to be critical in regulating EndMT [31]. When the Wnt signaling pathway is activated, the phosphorylation of β-catenin is inhibited and free β-catenin accumulates and translocates into the nucleus to modulate expression of EndMT regulatory genes, such as Twist, Snail and Slug [32–34]. Wnt/β-catenin signaling pathway participates in the EndMT of adult valvular endothelial cells [35] and human renal glomerular endothelial cell line [36]. It has also been shown that MALAT1 could promote the expression and nuclear accumulation of β-catenin in osteosarcoma [37], esophageal squamous cell carcinoma [38] and renal cell carcinoma [39].

In this study, we intended to elucidate whether MALAT1 participates in the regulation of ox-LDL-induced EndMT through the Wnt/β-catenin signaling pathway. We found that MALAT1 was dysregulated in arterial tissues from ApoE−/− mice with high-fat food-induced atherosclerosis and human umbilical vein endothelial cell line (HUVECs) stimulated with ox-LDL. Overexpression of MALAT1 had similar effect as ox-LDL on EndMT of HUVECs and nuclear translocation of β-catenin. MALAT1 knockdown rescued EndMT of HUVECs via partially reversing β-catenin nuclear accumulation triggered by ox-LDL. Collectively, these data highlight the importance of MALAT1 in the development and progression of atherosclerosis.

## Materials and methods

### Animals

Male C57BL/6 mice (8-week old) and ApoE−/− (B6/JNju-Apoeem1Cd82/Nju) mice were obtained from Nanjing Biomedical Research Institute of Nanjing University (Nanjing, China). C57BL/6 mice were fed with standard chow diet and were used as control group. ApoE−/− mice were fed with high-fat diet (HFD) containing 21% fat, 1.25% cholesterol for 16 weeks and were used as atherosclerosis model group. All animals were kept in rooms with constant room temperature (25 °C) and with 12 h light-dark cycle (light on from 8:00 AM to 8:00 PM). The animal experiment was performed in accordance with the Animal Ethics Committee of Hebei Medical University.

### Sample collection

The thoracic aorta was harvested from mice following 16 weeks of HFD (at 24 weeks of age). After humane euthanasia, the thorax was entered and the heart swiftly exposed. The left ventricle was punctured using a 20-gauge needle, and 1x PBS (20 ml) at 4 °C was infused at 2.5 ml/min. For H&E staining, the thoracic aorta was then fixed in situ by perfusing the heart with 20 ml of 4% paraformaldehyde in PBS. The thoracic aorta was then isolated and placed in 4% paraformaldehyde for 24 h. For real-time quantitative PCR, the thoracic aorta was harvested and preserved in liquid nitrogen. The blood was harvested from mice by removing the eyeball under anesthesia following 16 weeks of HFD (at 24 weeks of age). Serum was isolated from blood by centrifugation for 15 min at 3000 rpm/min at 4 °C and stored at − 80 °C until each assay was performed.

### Serum lipid levels

The serum total cholesterol (TC), triglyceride (TG), low-density lipoprotein cholesterol (LDL-C) levels were quantified by Hitachi 7080 automatic biochemical analyzer (Tokyo, Japan).

### Hematoxylin and eosin (H&E) staining

After gradient dehydration and transparency, the thoracic aorta was embedded with paraffin and subsequently sectioned at 4 μm thickness. Then, the sections were stained with Hematoxylin and Eosin Staining Kit (H&E) (Beyotime, Shanghai, China), and then photo-graphed with Leica DM6000B fully automatic biological microscope (Wetzlar, Germany).

### Cell culture and treatment

HUVECs were from Cell Bank of the Chinese Academy of Sciences (Shanghai, China) and were cultured in Dulbecco’s modified Eagle medium (Gibco BRL, Gaithersburg, MD, USA) containing 10% FBS (Gibco BRL, Gaithersburg, MD, USA), 2 mM glutamine, and 1% penicillin in a humidified atmosphere with 5% CO2 at 37 °C. HUVECs were treated with ox-LDL (Peking Union-Biology, Beijing, China) at different concentrations (10, 20, 40 μg/ml) for 48 h.

### Plasmid construction and cell transfection

For MALAT1 overexpression, a pcDNA3.1 expression vector containing full-length human MALAT1 (pcDNA-MALAT1) was constructed by Shanghai Integrated Biotech Solutions (Shanghai, China). For MALAT1 knockdown, three MALAT1-targeting siRNAs (MALAT1-siRNA1, 5′-GGUGGUGG UAUUUAGAUAATTUUAUCUAAA UACCACCACCTT-3′; MALAT1-siRNA2, 5′-GCGUCAUUUAAAGCCUAGUTTA CUAGGCUUUAAAUGACGCTT3’; MALAT1-siRNA3, 5′-GGGCUGACAUUAAC UACAATTUUGUAGUUAAUGUCA GCCCTT-3′) and a scrambled siRNA (sense 5′-UUCUCCGAACGUGUCACGUTT-3′, antisense 5′-ACGUGACACGUUCGGAGAA TT-3′) (Scramble) were designed and synthesized at GenePharma (Shanghai, China). Lipofectamine™ 3000 Transfection Reagent (Thermo Fisher, USA) was used. Real-time quantitative PCR was used to validate the efficiency of MALAT1 knockdown and overexpression. For knockdown of MALAT1, HUVECs were transfected with MALAT1 siRNA or negative control siRNA for 24 h before treatment with ox-LDL (40 μg/ml) for 48 h. For overexpression of MALAT1, HUVECs were transfected with MALAT1 pcDNA or MALAT1 expressing vector for 48 h.

### Cell morphology analysis

For observing cell morphology, cellular F-actin was stained with rhodamine phalloidin (Thermo Fisher, USA), and nuclei were stained with DAPI Fluoromount-G® (Southern Biotech, Birmingham, USA). Then the cells were imaged using a confocal laser scanning microscopy (ZEISS, Oberkochen, Germany).

### Real-time quantitative PCR

Total RNA was extracted from fresh frozen thoracic aorta or cells collected at indicated time using Eastep® Super Total RNA Extraction Kit (Promoga, Beijing, China). The cDNA was prepared using the PrimeScript™ RT Reagent Kit with gDNA Eraser (Takara Clontech, Kyoto, Japan). Real-time quantitative PCR was performed in triplicates using TB Green™ Premix Ex Taq™ (Takara Clontech, Kyoto, Japan) on applied biosystems 7300 Real-Time PCR system (Bio-rad, CA, USA). The primers were purchased from Sangon Biotech CO., Ltd. (Shanghai, China). The sequences were shown in Table 1. β-actin was used as internal control. Relative expression of each gene was calculated using the 2^-ΔΔCt^ method [40].

**Table 1:** The sequences for the primers

Species	Gene (Accession No.)	Primer direction	Sequence(5’-3’)
Mouse	MALAT1 (NR_002847)	Forward	5′-GATAGCCCAGGAAAGAGTGC-3′
		Reverse	5′-TCACCACCACATCCGTATG-3’
Mouse	β-actin (NM_007393.2)	Forward	5′-AGCCTTCCTTCTTGGGTATG-3’
		Reverse	5′-GGTCTTTACGGATGTCAACG-3’
Human	MALAT1 (NR_002819)	Forward	5′-AAAGCAAGGTCTCCCCACAAG-3’
		Reverse	5′-GGTCTGTGCTAGATCAAAAGGCA-3’
Human	CD31 (NM_000442.4)	Forward	5′-CACTTCTGAACTCCAACAACG-3’
		Reverse	5′-GGACACTTGAACTTCCGTG-3’
Human	vWF (NM_000552.3)	Forward	5′-ATGGTTCTGGATGTGGCGT-3’
		Reverse	5′-TTGCTCCTGTTGAAGTCGG-3’
Human	α-SMA (NM_001141945)	Forward	5′-TGAAGAGCATCCCACCCT-3’
		Reverse	5′-ATAGAGAGACAGCACCGCC-3’
Human	Vimentin (NM_003380.4)	Forward	5′-AAATGGCTCGTCACCTTCG-3’
		Reverse	5′-AGAAATCCTGCTCTCCTCGC-3’
Human	β-actin (NM_001101.3)	Forward	5′-GGTCATCACCATTGGCAA-3’
		Reverse	5′-GAGTTGAAGGTAGTTTCGTGGA-3’

### Western blot

The total protein was extracted from cells with ice-cold lysis buffer supplemented with protease inhibitor cocktail (Merck, Germany) and PMSF. The nuclear protein was extracted with Nuclear and Cytoplasmic Protein Extraction Kit (Beyotime, Shanghai, China). The protein concentration was determined with Detergent Compatible Bradford Protein Assay Kit (Beyotime, Shanghai, China). Proteins were separated on SDS-PAGE gels and transferred onto nitrocellulose membrane (Life Technologies, USA). After blocking with Odyssey® blocking buffer (PBS) (LI-COR, Lincoln, USA), the membranes were incubated overnight with the primary antibodies of CD31 (ab28364,1:500), vWF (ab6994,1:500), α-SMA (ab32575,1:1000), vimentin (ab92547,1:1000), Histone H3 (ab1791,1:1000), β-catenin (ab32572,1:5000) and GAPDH (ab181602,1:10000) (abcam, Cambridge, UK). After that, IRDye 800CW Goat anti-Rabbit IgG (H + L) Secondary Antibody (926–32,211, 1:10000), IRDye® 680LT Goat anti-Rabbit IgG (H + L) Secondary Antibody (926–68,021) (LI-COR, Lincoln, USA) were added and incubated for 1 h at room temperature. Then the band intensities were scanned by Odyssey (LI-COR, Lincoln, USA) and normalized to GAPDH or Histone H3.

### Immunofluorescence analysis

After ox-LDL treatment or transfection, immunostaining of HUVECs was performed. Cells were fixed with 4% polyoxymethylene for 10 min at room temperature, 10 min at 4 °C and methanol for 5 min, then blocked with 1% BSA for 1 h at room temperature and incubated with primary antibodies of CD31 (ab28364,1:20), vWF (ab6994,1:400), α-SMA (ab32575,1:500), vimentin (ab92547,1:300), and β-catenin (ab32572,1:250) (Abcam, Cambridge, UK) overnight at 4 °C. After washing, Goat Anti-Rabbit IgG H&L (DyLight® 488) (ab96899, 1:200) was added and incubated for 1 h at room temperature, followed by nuclei staining with DAPI Fluoromount-G® (Southern Biotech, Birmingham, USA). Then the cells were imaged using a confocal laser scanning microscopy (ZEISS, Oberkochen, Germany).

### Statistical analysis

All graphs were created using Graphpad Prism software. All data were analyzed using SPSS version 19.0 software (SPSS lnc) and presented as mean ± SD (standard deviation). The two-tailed Student’s t-test was used to evaluate differences between two groups. Results from multiple groups were compared using one-way ANOVA test. Correlation analyses between variables were performed using the Pearson rank correlation test. *P* < 0.05 was accepted as statistically significant.

## Results

### MALAT1 is upregulated in mouse model of atherosclerosis

**Fig. 1:**
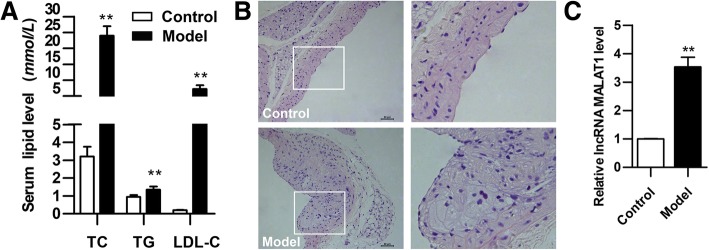
Changes of serum lipid, histology and MALAT1 in mice**.** *ApoE*^−/−^ mice were fed with HFD for 16 weeks, and normally fed C57B/6 mice were used as control. **a** Serum lipid levels of the two groups were detected by automatic biochemical analyzer (*n* = 9, **P < 0.01, versus control group). **b** H&E staining of vessel wall. Magnification, 200x. **c** Level of MALAT1 in aortas tissues as determined by qRT-PCR (*n* = 3, **P < 0.01, versus control group)

Serum TC, TG and LDL-C levels in mice after 16-week HFD diet were measured. As shown in Fig. 1a, serum TC, TG and LDL-C levels was increased significantly when compared with the control group of mice. The aortal tissues were stained with H&E. Atherosclerotic plaques were observed clearly in the aortic sections of model group, but not that of control group (Fig. 1b). Next, we detected MALAT1 expression in aortas upon atherosclerosis induction. We found that MALAT1 level was remarkably up-regulated in aortas of model group compared with that of control group (Fig. 1c). These results indicate that with the dyslipidemia, the level of lncRNA MALAT1 is elevated in the arterial tissue of atherosclerotic mice.

### MALAT1 is upregulated during ox-LDL induced EndMT of HUVECs

**Fig. 2:**
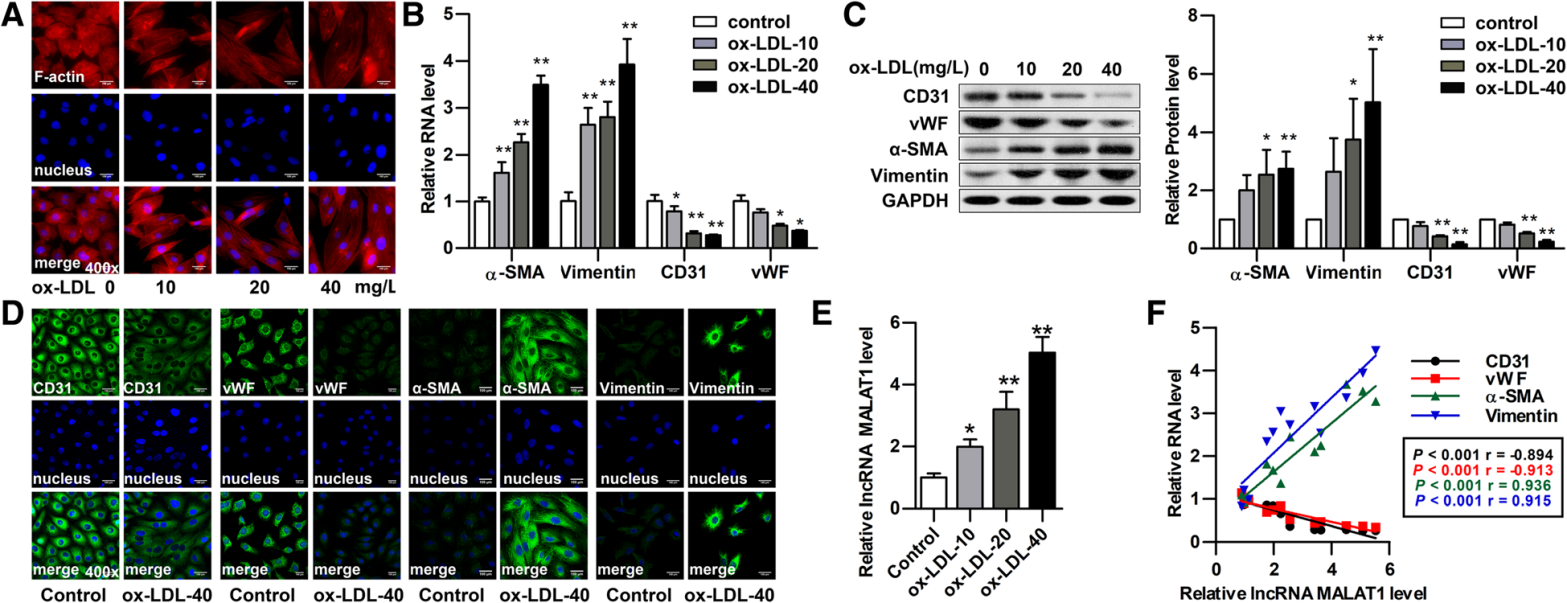
Changes of cellular morphology, EndMT markers and MALAT1 in HUVECs. HUVECs were treated with ox-LDL at different concentrations (10, 20, 40 μg/ml) for 48 h. **a** Cellular F-actin was stained with rhodamine phalloidin. Nuclei were stained with DAPI Fluoromount-G®. Cells were observed under fluorescence microscopy. Magnification, 400x. **b-d** Endothelial markers (CD31, vWF) and mesenchymal markers (α-SMA, vimentin) were detected by qRT-PCR (B), Western blot (C) (n = 3, *P < 0.05, **P < 0.01, versus control group) and immunofluorescence analysis (D). Magnification, 400x. **e** MALAT1 expression was detected by qRT-PCR (n = 3, *P < 0.05, **P < 0.01 versus control group). The relative levels of α-SMA, vimentin, CD31, vWF and MALAT1 in ox-LDL groups were normalized to those in control group, respectively. **f** Pearson correlations between MALAT1 and α-SMA mRNA, vimentin mRNA, CD31 mRNA, vWF mRNA

Next, we investigated the expression and role of MALAT1 in HUVECs. HUVECs were treated with ox-LDL at different concentrations (10, 20, 40 μg/ml) for 48 h. After incubation, the HUVECs underwent an EndMT transition with the increase of ox-LDL, as confirmed by its morphological change to a spindle-shape (Fig. 2a), decreased expression of endothelial markers CD31 and vWF along with enhanced expression of mesenchymal markers α-SMA and vimentin (Figs. 2b–d). MALAT1 was then detected in these HUVECs by qRT-PCR, and its expression was significantly increased in ox-LDL-treated cells with increased ox-LDL concentration (Fig. 2e). As shown is Fig. 2f, there was significantly positive correlation between α-SMA mRNA and MALAT1, or vimentin mRNA and MALAT1 (two-sided Pearson correlation, rα-SMA = 0.936, rvimentin = 0.915, *P* < 0.001), and significantly negative correlation between CD31 mRNA and MALAT1, or vWF mRNA and MALAT1 (two-sided Pearson correlation, rCD31 = − 0.894, rvWF = − 0.913, *P* < 0.001). These results indicate that MALAT1 may contribute to ox-LDL-induced EndMT of HUVECs, and 40 μg/ml ox-LDL was used in following studies.

### Knockdown of MALAT1 attenuates ox-LDL-induced EndMT in HUVECs

**Fig. 3:**
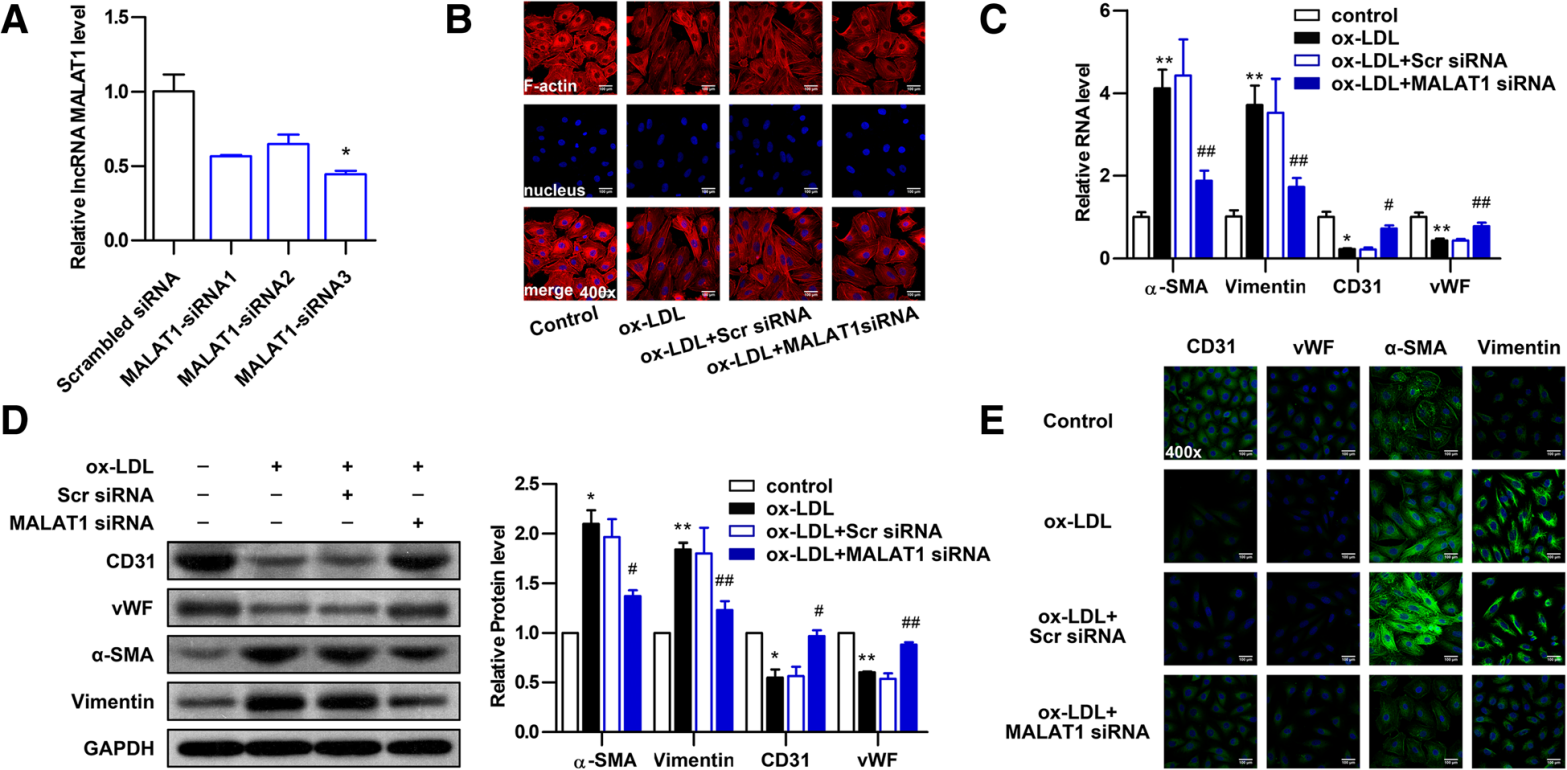
Knockdown of MALAT1 attenuates ox-LDL-induced EndMT in HUVECs. **a** HUVECs were transfected with MALAT1-siRNA1, MALAT1-siRNA2, MALAT1-siRNA3 or scramble control (scr) for 24 h. The MALAT1 levels were determined by qRT-PCR (n = 3, *P < 0.05, versus negative control group). **b** The effect of MALAT1-siRNA on cell morphology induced by ox-LDL (40 μg/ml) was observed through rhodamine phalloidin-stained F-actin. Magnification, 400x. **c-e** The effect of MALAT1-siRNA3 on the expression of endothelial markers (CD31, vWF) and mesenchymal markers (α-SMA, vimentin) were detected by qRT-PCR (**c**), Western blot (**d**) (*n* = 3, *P < 0.05, **P < 0.01 versus control group, ^#^*P* < 0.05, ^##^*P* < 0.01 versus ox-LDL group) and immunofluorescence analysis (**e**). Magnification, 400x

To identify whether silencing of MALAT1 could abolish EndMT of HUVECs induced by ox-LDL, three MALAT1-siRNAs (MALAT1-siRNA1, MALAT1-siRNA2, MALAT1-siRNA3) were used to knock down MALAT1. The knockdown efficiency of MALAT1-siRNA3 was optimal and thus MALAT1-siRNA3 was used for further experiments (Fig. 3a). After ox-LDL treatment, Cells transfected with MALAT1-siRNA3 displayed cobblestone-like endothelial appearance, while cells transfected with scramble control maintained spindle-like mesenchymal phenotype (Fig. 3b). Next, the markers of EndMT were detected by qRT-PCR, Western blot and immunofluorescent staining. Upon ox-LDL treatment, cells transfected with MALAT1-siRNA displayed significantly decreased mRNA and protein levels of the mesenchymal markers α-SMA and vimentin along with increased levels of the endothelial markers CD31 and vWF compared with cells transfected with scramble or untransfected cells (Figs. 3c-e). These data suggest that knockdown of MALAT1 attenuates ox-LDL-induced EndMT in HUVECs.

### Overexpression of MALAT1 promotes EndMT in HUVECs

**Fig. 4:**
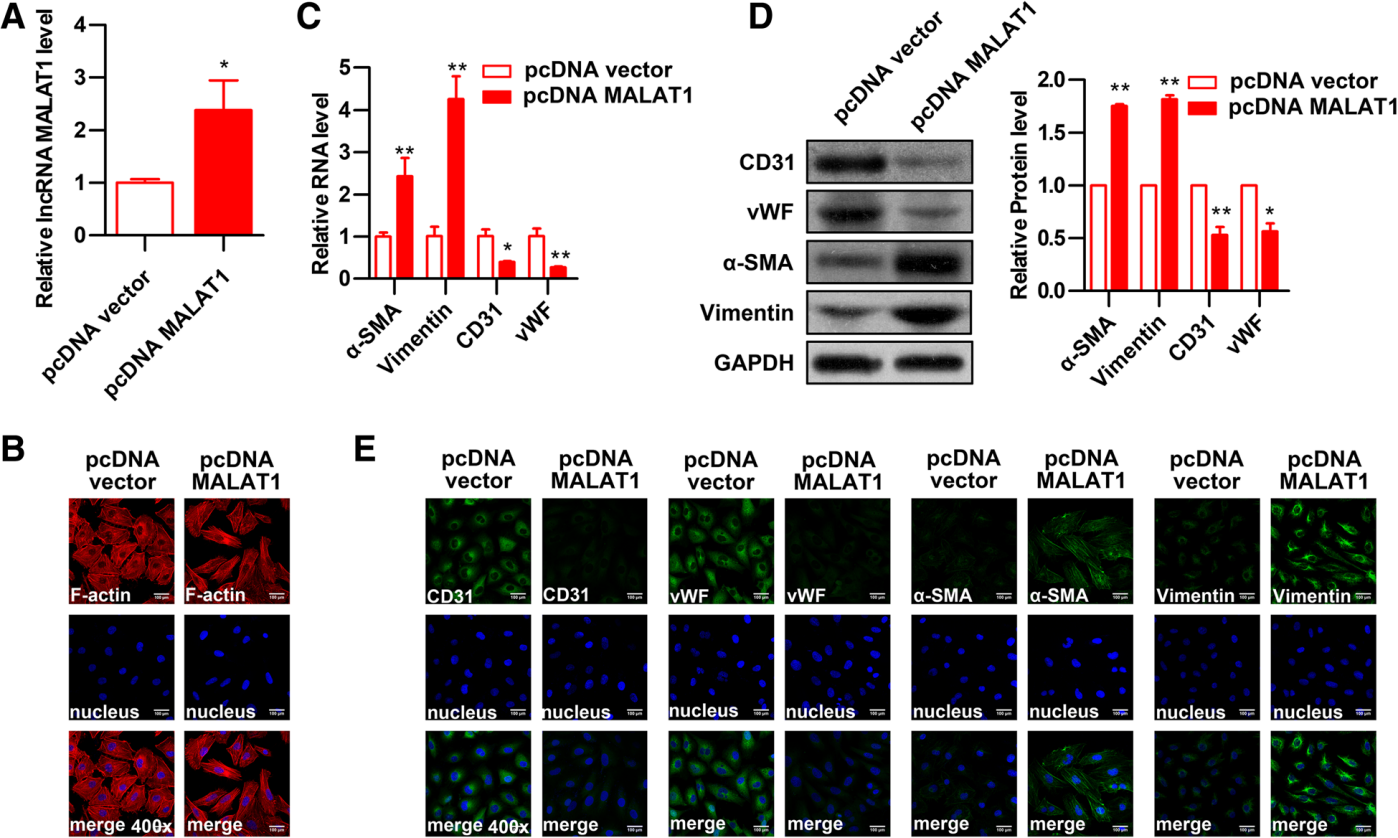
The effect of MALAT1 overexpression on EndMT of HUVECs. Full-length human MALAT1 cDNA plasmid (pcDNA-MALAT1) was constructed and transfected into HUVECs. The empty vector was used as negative control. **a** MALAT1 expression was detected by qRT-PCR (n = 3, *P < 0.05, versus pcDNA vector group). **b** The effect of MALAT1 overexpression on cell morphology induced by ox-LDL (40 μg/ml) was observed through rhodamine phalloidin-stained F-actin. Magnification, 400x. **c-e** The effect of MALAT1 overexpression on the expression of endothelial markers (CD31, vWF) and mesenchymal markers (α-SMA, vimentin) were detected by qRT-PCR (**c**), Western blot (**d**) (*n* = 3, *P < 0.05, **P < 0.01 versus pcDNA vector group) and immunofluorescence analysis (**e**). Magnification, 400x

To further determine the effect of MALAT1 on EndMT of HUVECs, MALAT1 was overexpressed in HUVECs. The qRT-PCR assay was applied to test the level of MALAT1 and the result showed that MALAT1 expression in pcDNA-MALAT1 transfected HUVECs was increased to 2.4 folds compared to control group (Fig. 4a). MALAT1-overexpressed cells showed more cells with spindle-like mesenchymal phenotype upon ox-LDL treatment compared with control cells (Fig. 4b). Analysis of EndMT biomarkers CD31, vWF, α-SMA, and vimentin by qRT-PCR, Western blot and immunofluorescent staining showed that pcDNA-MALAT1 transfection significantly decreased mRNA and protein levels of the endothelial markers CD31 and vWF along with significantly increased levels of the mesenchymal markers α-SMA and vimentin compared with cells transfected with pcDNA vector (Figs. 4c-e). These results suggest that MALAT1 plays an important role in the process of EndMT of HUVECs induced by ox-LDL.

### Knockdown of MALAT1 inhibits ox-LDL-induced activation of Wnt/β-catenin pathway

**Fig. 5:**
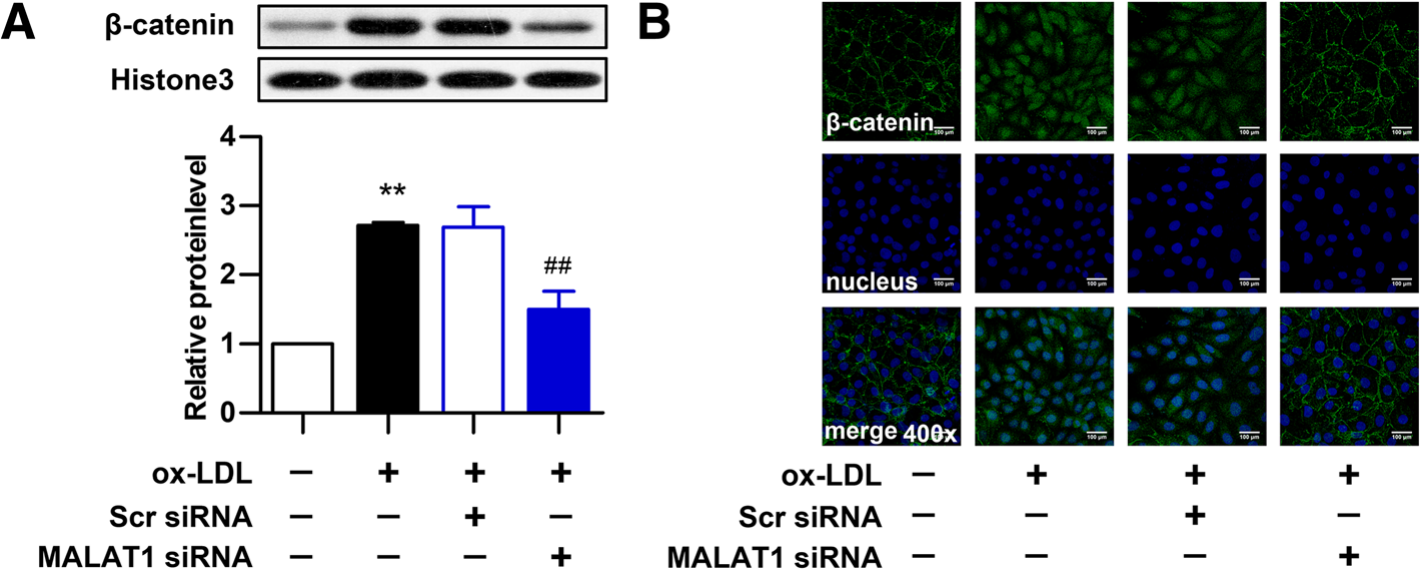
Knockdown of MALAT1 inhibits ox-LDL-induced β-catenin translocation. HUVECs were transfected with MALAT1-siRNA or scramble control (scr) for 24 h. **a** The protein expression of β-catenin in nucleus (n = 3, *P < 0.05, versus control group, ^#^*P* < 0.05, versus model group) was analyze by Western blot. **b** The location of β-catenin was detected by immunofluorescence analysis. Magnification, 400x

Ox-LDL can upregulate the expression of β-catenin in cell nuclei, a hallmark of Wnt/β-catenin pathway activation [41]. Therefore, MALAT1 was knocked down to investigate the role of MALAT1 in ox-LDL induced Wnt/β-catenin activation in HUVECs. The expression and location of β-catenin was examined by western blot (Fig. 5a) and immunofluorescence analysis (Fig. 5b). MALAT1 knockdown reversed the increased expression and nucleus translocation of β-catenin induced by ox-LDL, indicating that MALAT1 knockdown inhibits ox-LDL-induced activation of Wnt/β-catenin signaling in HUVECs.

### Overexpression of MALAT1 promotes activation of Wnt/β-catenin pathway by ox-LDL

**Fig. 6:**
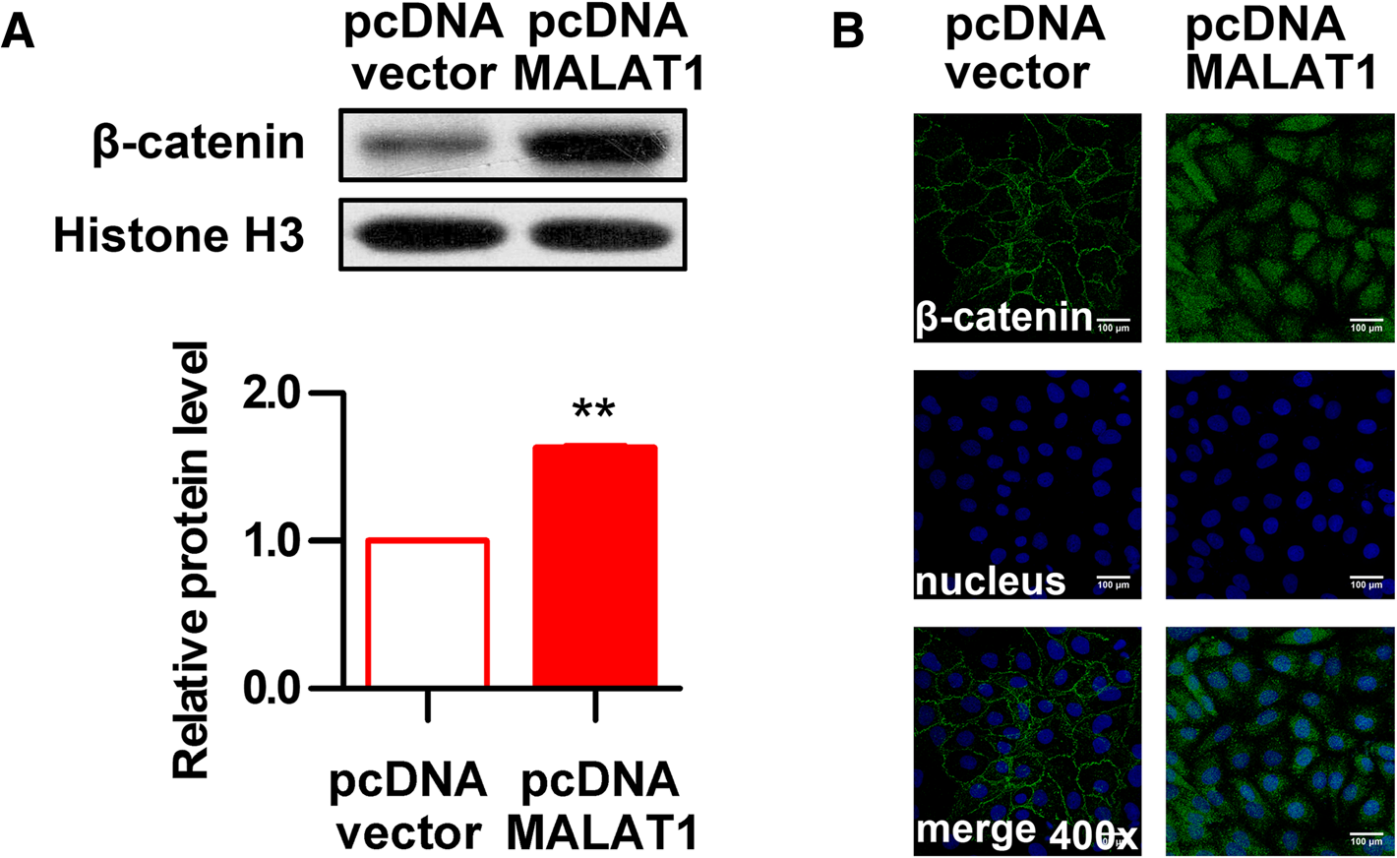
Overexpression of MALAT1 promotes activation of Wnt/β-catenin pathway. HUVECs were transfected with pcDNA-MALAT1 or pcDNA vector for 48 h. **a** The protein level of β-catenin in nucleus (*n* = 3, *P < 0.05, versus pcDNA vector group) was analyze by Western blot. **b** The location of β-catenin was detected by immunofluorescence analysis. Magnification, 400x

To further analyze the effect of MALAT1 on the activation of Wnt/β-catenin signaling, the expression and location of β-catenin was examined through western blot (Fig. 6a) and immunofluorescence analysis (Fig. 6b) after MALAT1 overexpression and ox-LDL induction. The results showed that HUVECs transfected with MALAT1-overexpressing plasmid increased β-catenin level in the cell nucleus compared with control cells, suggesting that MALAT1 overexpression further promotes ox-LDL-induced Wnt/β-catenin signaling pathway activation.

## Discussion

There is increasing evidence that EndMT plays important roles in the occurrence and development of atherosclerosis [6] and promoting the instability of plaques [5]. When EndMT occurs, endothelial cells present the shape and properties of mesenchymal cells (smooth muscle cells, fibroblasts), including migration, proliferation, secretion of extracellular matrix (fibronectin, collagen, et al) and matrix metalloproteinases, and, expression of various leukocyte adhesion molecules [3], all of which are related to the formation, progression and instability of atherosclerotic plaques. Pathologic intimal thickening in human atherosclerosis is formed by dispersion of intimal smooth muscle cells and extracellular accumulation of plasma-derived lipids [42]. EndMT results in a loss of barrier function, that is, increased permeability, which is conducive to the passage and deposition of lipids [43], and EndMT is one of the sources of vascular smooth muscle cells in vascular intima [3]. The role of EndMT in atherosclerosis is very clear, but its mechanism remains to be further studied.

Extensive evidence shows that MALAT1 plays critical roles in coronary atherosclerotic heart disease [44]. For instance, MALAT1 was upregulated in patients with unstable angina pectoris [45], but another study showed that MALAT1 was less expressed in atherosclerotic coronary artery plaques compared to the non-atherosclerotic internal artery, which is histopathologically similar to coronary artery in terms of structural layers but atherosclerosis-resistant [15]. Moreover, rs619586AG/GG genotypes in MALAT1 were reported to protect against the occurrence of coronary atherosclerotic heart disease [46]. In the present study, MALAT1 is upregulated in arterial tissues of in HFD-fed ApoE−/− mice. This is in line with previous studies, demonstrating that MALAT1 is upregulated in macrophages [24] and HUVECs [47] treated with ox-LDL, and in proliferative vascular smooth muscle cells [22], all of which are critical cells for atherosclerosis. Then, it has been shown that MALAT1 plays a significant role in the process of EMT and EndMT. MALAT1 promotes EMT of nasopharyngeal carcinoma cells through de-repressing Capn4 by sponging miR-124 [48] and mediates TGF-β1-induced EndMT by regulating TGFBR2 and SMAD3 through miR-145 in endothelial progenitor cells (EPCs) [25]. Thus, we performed further in vitro studies to elucidate the role of MALAT1 in EndMT process of atherosclerosis.

LDL-C, especially ox-LDL, plays a central role in the development of atherosclerosis, and subclinical atherosclerosis remains common in individuals with LDL-C < 70 mg/dl [49]. Ox-LDL activates the receptor of ox-LDL in endothelial cells [10] to up-regulate stimulators of EndMT, such as inflammatory cytokines [11], ROS [12] and TGF-β [13]. Moreover, ox-LDL can promote EMT of prostate cancer cells [50] and human renal proximal tubular epithelial cells [51]. Recent study showed that ox-LDL induced EndMT by stabilizing Snail in human aortic endothelial cells [52]. In the present study, the morphology of HUVECs changed from cobblestone-like shape to spindle shape after induction by ox-LDL, accompanying with a decrease of endothelial markers CD31 and vWF, and an increase of mesenchymal markers α-smooth muscle actin (α-SMA) and vimentin. These data suggest that ox-LDL could induce EndMT in HUVECs. Furthermore, there was a significant linear correlation between the change of MALAT1 and EndMT markers, suggesting a link between MALAT1 and EndMT of HUVECs induced by ox-LDL. Next, MALAT1 was knocked-down or overexpressed to detect whether MALAT1 participates in the process of ox-LDL-induced EndMT. As expected, MALAT1 silencing reversed the changes of morphology and EndMT markers in HUVECs treated with ox-LDL. In contrast, ectopic MALAT1 expression could induce the expression of CD31 and vWF, and inhibited the expression of α-SMA and Vimentin, with slightly changed morphology of HUVECs. These data suggested that MALAT1 was involved in ox-LDL-induced EndMT.

Wnt/β-catenin is one of the signaling pathways involved in EndMT [53]. When Wnt/β-catenin signaling pathway is activated, β-catenin is translocated into the nuclei to interact with transcription factors from the T-cell factor / leucocyte enhancer factor family and to activate transcription of Wnt responsive genes, leading to the occurrence of EndMT [54]. Here in this study, Wnt/β-catenin pathway was activated by ox-LDL, as demonstrated by increased protein expression of β-catenin in nuclei. The expression and nuclear transposition of β-catenin induced by ox-LDL was inhibited by MALAT1 knockdown while promoted by MALAT1 overexpression. So it can be concluded that MALAT1 may modulate ox-LDL-induced EndMT partially through the Wnt/β-catenin signaling pathway.

## Conclusions

This study demonstrates that MALAT1 is dysregulated in arterial tissues from atherosclerotic mice and HUVECs treated with ox-LDL, and uncovers the important role of MALAT1 in promoting ox-LDL induced HUVEC EndMT in vitro, which is dependent on Wnt/β-catenin signaling pathway. Thus, these findings might connect the ox-LDL-induced EndMT to its notorious role in atherosclerosis and highlight the importance of MALAT1 in the development of atherosclerosis.

## Abbreviations

EMT: Epithelial-to-mesenchymal
EndMT: Endothelial-to-mesenchymal transition
HFD: High-fat diet
HUVECs: Human umbilical vein endothelial cell line
LDL-C: Low-density lipoprotein cholesterol
MALAT1: Metastasis-associated lung adenocarcinoma transcript 1
ox-LDL: Oxidized low-density lipoprotein
TC: Total cholesterol
TG: Triglyceride
